# Evaluation of the self-sampling for cervical cancer screening in Bolivia

**DOI:** 10.1186/s12889-019-6401-5

**Published:** 2019-01-17

**Authors:** Gustavo Allende, Pedro Surriabre, Leyddy Cáceres, Diego Bellot, Neli Ovando, Andrea Torrico, Pamela Calle, Carla Ascarrunz, Sophie Alexander, Michel Bossens, Véronique Fontaine, Patricia Rodríguez

**Affiliations:** 1 Laboratorio Virología, Facultad de Medicina, Universidad Mayor de San Simón, Cochabamba, Bolivia; 2 Unité de Microbiologie Pharmaceutique et Hygiène, Faculté de Pharmacie, Université Libre de Bruxelles (U.L.B.), CP205/2, Campus Plaine, Boulevard du Triomphe, 1050 Brussels, Belgium; 3 Institute of Social Sciences Research (INCISO). Faculty of Social Sciences, Universidad Mayor de San Simón, Cochabamba, Bolivia; 4 Ecole de Santé Publique, Université Libre de Bruxelles (U.L.B.), CP596, route de Lennik 808, 1070 Brussels, Belgium; 5 Research laboratory in human reproduction, Campus Erasme, Faculty of Medicine, Université Libre de Bruxelles, Brussels, Belgium

**Keywords:** HPV screening, HPV self-sampling, Coverage, Acceptability, Cervical cancer

## Abstract

**Background:**

Incidence and mortality rates of cervical cancer in Bolivia are the highest in Latin America. Vaginal cell self-sampling can improve screening coverage. Information on common reasons for low screening coverage and preferences for future screening are essential to reduce cervical cancer incidence. We aimed to evaluate the knowledge about human papillomavirus (HPV) and cervical cancer of Bolivian women from urban, peri-urban and rural areas of Cochabamba and to determine their degree of acceptability and confidence towards vaginal HPV self-sampling. In addition, we assessed the impact of self-sampling on cervical cancer screening coverage in a selected peri-urban area.

**Methods:**

We gathered information from women living in urban, peri-urban and rural areas of Cochabamba province in Bolivia using two different structured questionnaires. In Survey1, we collected information from 222 women about their knowledge on HPV and cervical cancer. In Survey 2, the acceptance and confidence towards vaginal HPV self-sampling compared to the physician-sampling was assessed in 221 women. A non-probabilistic stratified sampling by areas was carried out for the two questionnaires. In the third phase of the study, we determined the impact of HPV self-sampling collection on screening coverage in a peri-urban area of Cochabamba.

**Results:**

Bolivian women knew little or nothing about cervical cancer and HPV infection in all areas. They all found self-sampling collection easier to perform (86.9 to 93.2%) and more comfortable (79.4 to 83.3%) compared to physician sampling. Sampling accuracy to detect cervical cancer was probably higher in their point of view when it was taken by physician (35.1 to 63.5%). However in rural areas women preferred self-sampling. Accordingly, the campaign of vaginal HPV self-sampling in this peri-urban area increased screening coverage, reaching in three months the annual rate average.

**Conclusions:**

The knowledge about cervical cancer and HPV infection is poor in Bolivia. Despite greater acceptance of the vaginal HPV self-sampling in all areas, women kept greater confidence in the screening performed by the gynecologist although HPV self-sampling improved coverage rate.

**Electronic supplementary material:**

The online version of this article (10.1186/s12889-019-6401-5) contains supplementary material, which is available to authorized users.

## Background

The development of cervical cancer depends on high-risk human papillomavirus (HR-HPV) persistent infection in the uterine cervix [1]. The 2–10 year transformation process leading to invasive cancer provides ample opportunity to detect, prevent and cure true precursor lesions [1]. Although cervical cancer is widely preventable, it is the fourth most common cancer among women throughout the world, being a real public health issue, especially in developing countries, as 85% deaths occur in low and middle income countries [2]. The situation in Bolivia is particularly alarming as the incidence, being 47 per 100,000 women, is estimated to be the highest in Latin America with a mortality rate of 21 per 100,000 women^1^ (standardized incidence and mortality rates by age) [2].

Cervical cancer-related deaths have nevertheless declined significantly in developed countries because of extensive cytology screening. However, similar initiatives in developing countries have not been equally successful because of the complexity of the required elements, such as laboratory expertise, depending on high operating costs [3, 4]. It is now well known that the HR-HPV screening test has marked advantages over cytology screening test, with sensitivity of about 90% for detecting high-grade intraepithelial neoplasms and high negative predictive value [5, 6].

Prevention of cervical cancer in Bolivia is primarily based on Papanicolaou smear cytology test (Pap) and more recently on visual inspection under acetic acid (VIA) [7, 8]. Screening, offered by the first level of care, responsible for prevention, is available free of charge, for sexually active women until 64 years old [7]. Nevertheless, Pap smear coverage, from 2005 to 2016, do not exceed 16.6% and coverage of VIA in 2015 and 2016 does not exceed 19% [7, 8].

The health personnel estimated that 50 to 80% of Pap screened women were lost to follow-up, mainly because of delays in result delivery [3].

Bolivia’s Ministry of health has introduced the HPV vaccine in 2016 as an alternative to reduce the incidence of cervical cancer. In 2017, 80% of the target population (girls between 10 and 12 year old) were vaccinated with the first dose [9].

Bolivia is characterized by significant disparities between rural and urban areas, including education and access to basic health services as some of the variables contributing to this difference [10, 11]. Although these differences have been reduced in recent years, the inequalities still persist among the poorest population [12, 13]. Poor coverage of the Pap smear, Pap poor quality, low follow-up of Pap positive cases, lack of information on cervical cancer prevention, poor human resources in health and low credibility in the health system, besides economic, cultural, and social barriers have been identified as the main factors involved in the high cervical cancer incidence in Bolivia [3, 14]. Large coverage disparity has been observed when stratified by area of residence, being much lower in rural areas than in urban areas and increasing the risk of dying from cervical cancer up to three fold in dispersed rural areas. This is linked to various factors, including low educational, cultural and economic level and limited access to health services, diagnostic tests and treatment [15].

The screening coverage rate is a key component in cervical cancer prevention programs. Vaginal self-testing of HR-HPV could reduce screening barriers for those women and therefore increase their screening coverage [16–18]. Indeed, self-collection is easy to perform, provides privacy, is less embarrassing and more comfortable to patients compared to samples collected by health personnel [19, 20]. Furthermore, self-collected samples have been shown to have sensitivity similar to that of samples collected by physicians [21]. We previously reported that a simple, cheap and transport safe method based on the smearing of vaginal and cervical cells on a glass slide (dry samples) using cotton swab gave satisfactory results to detect HR-HPV DNA, providing similar HR-HPV detection results as the physician collected samples [22].

The objectives of this study were to obtain information among the Bolivian women about their level of knowledge on HPV and cervical cancer and to evaluate the degree of the vaginal self-sampling test acceptability and confidence in comparison to gynecologist sampling at urban, peri-urban and rural areas, in order to evaluate the need for an adapted strategy within each three geographical areas, potentially corresponding to a characteristic population. Furthermore, this study assessed the impact of the HR-HPV self-sampling test on screening coverage in a restricted Bolivian peri-urban area.

## Methods

This cross sectional study is based on the analysis of two surveys with a non-probabilistic stratified sampling by geographical areas (including disparities in access to education, health system and financial income). The evaluation of coverage impact through self-sampling was performed in a peri-urban area, with a mixed population (in regards to the disparities already mentioned above). The Bio-ethical committee of “Universidad Mayor de San Simon” approved the study protocol (October 30th, 2014). The signed informed consent was an indispensable requirement for the inclusion of patients. Pregnant women over 20 weeks, calculated on the basis of last menstrual period, and women with hysterectomy were excluded from this study. This study was divided in three phases. For surveys, a person speaking Quechua and Spanish was available to read and help to fill the survey files in case of analphabetism.

### Survey 1: Cervical cancer knowledge assessment

In the first phase of the study (survey 1), we obtained information from 222 women about their knowledge on HPV and cervical cancer and their prevention. The target population was women from the urban (Central neighborhood, *n* = 96), the peri-urban (surrounding area *n* = 66) and the rural (Chapare, *n* = 60) areas with a range of age between 20 and 64 years. One structured questionnaire was conducted with four multiple selection questions in the three geographic areas mentioned above to determine the degree of knowledge about cervical cancer and HPV, (see Additional file 1). According to the number of correct answers, the categories were assigned: “poor knowledge”, or “good knowledge”. In addition, data on level of education and age were obtained.

The women surveyed in urban areas came from professional and community associations. Women from the peri-urban and rural areas were surveyed at home or on their workplace.

### Survey 2: Assessment of acceptance and confidence in self-sampling

The second phase of the study (survey 2) assessed 221 women through eight closed-ended questions after self-sampling and physician-sampling to determine their acceptance and confidence towards vaginal HPV self-sampling. The target population was women from the urban (Central neighborhood, *n* = 74), the peri-urban (surrounding area *n* = 63) and the rural (Chapare, *n* = 84) areas, with a range of age between 25 to 64 years.

The HPV self-sampling provided kit, previously tested [22], consisted of gloves, sterile cotton swab, and card with glass slides (see Additional file 2). It also included a private data form, instructions and an informed consent form. The instructions given were as follows: put on the gloves with both hands, take the envelope with the glass slide, open it and place it on a clean surface. Position yourself in a comfortable position, remove the cotton swab from the cotton-free end of the envelope, taking care not to touch any surface and insert the swab into the vagina until you feel a slight resistance. Then, rotate the swab three times, remove it and extend the sample by rubbing the end of the cotton on the surface of the glass slide. The sample on the glass slide should then be air dried for a few minutes before closing the envelope and placing it inside the plastic bag to be delivered to volunteers or health personnel. The description of the way that the physician took the sample is the following: first the physician placed the speculum in the vagina, then it took the sample using a cytobrush.

Women from the urban, peri-urban and rural areas were surveyed in the attended health centers.

### Evaluation of HPV self-sampling on screening coverage

In the third phase, we made interventions to promote cervical cancer screening in the southern district 9 (peri-urban area that congregates a mixed rural urban population). This was carried out through campaigns with the participation of volunteers (health personnel, and medical students and students in sociology from University Mayor San Simon). All of them were trained for three days to give correct information on cervical cancer, HPV and on the self-sampling technique and procedure. Two strategies to promote and collect self-samplings were performed: 1) direct contact with the population through visits at home, informing and offering the self-sampling device, 2) installation of tents with isolated cubicles in populated areas, generating a favorable environment for self-sampling. This was carried out during 9 one-day campaigns over a three month period. Cervical cancer screening was also promoted for 8 months in the primary Health Centers of District 9 Cochabamba, starting four months before the beginning of the self-sampling campaigns. The device for the vaginal and cervical cell self-collection was the same as in phase 2. The results in the coverage achieved through this intervention were estimated for twelve months and compared to the cytology coverage obtained in the previous year.

### HR-HPV DNA detection

All cervico-vaginal samples in this study were analyzed for HR-HPV DNA within two weeks after they were collected. The procedure for DNA extraction from samples transported and conserved on glass slides has been described previously [22]. Only DNA samples positive in beta-globin PCR (considered as having good DNA quality) were further analyzed for HR-HPV DNA. The presence of HR-HPV DNA was assessed by the PCR GP5+/6+ followed by an enzyme immune-assay (GP-EIA) [23].

### Statistics

Statistical analyses were performed using hypothesis test for proportion (Chi-square). The null hypothesis was that there are no differences between the responses of the urban, peri-urban and rural areas, (*P* value greater than 0.05), assuming that differences in education between the geographic residences of women are not influencing the survey responses. This null hypothesis is justified to detect any difference between areas on the survey responses.

The coverage was calculated considering the target population and women screened by Pap one year earlier. The projection estimation of the coverage on 12 months was carried by taking into account the maintenance of similar efficacy for one year (rule of three).

## Results

### Age and education characteristics of the studied population

In the first and second phase of the study, most of the population (83% for the survey 1 and 95% for the survey 2) was in the range of 20 to 49 years (data not shown). As expected, the level of education of the survey 1 population decreased in relationship with the distance to the urban region (Table 1).

**Table 1:** Woman distribution by education and level of knowledge about cervical cancer in survey 1

Survey 1 (*N* = 222)													
	Total		Primary School			High School		University		others		No data	
Region	*N*	%	*n*	%		*n*	%	*n*	%	*n*	%	*n*	%
Urban	96	100	2	2.1		15	15.6	63	65.6	13	13.5	3	3.1
Periurban	66	100	23	34.8		16	24.2	19	28.8	3	4.5	5	7.6
Rural	60	100	49	81.7		6	10.0	3	5.0	2	3.3	0	0
Total sites	222	100	74	33.3		37	16.6	85	38.1	18	8.1	8	3.6
*P* value			0.000			0.08		0.000					
	Total		What do you know about CC?					What do you know about prevention of CC?					
Region	*N*	%	good knowledge			bad knowledge		good knowledge			bad knowledge		
			*n*		%	*n*	%	*n*	%		*n*	%	
Urban	96	100	34		35.4	62	64,6	31	32.3		65	67.3	
periurban	66	100	14		21.2	52	78,8	8	12.0		58	88.0	
Rural	60	100	1		1.7	59	98,3	1	1.7		59	98.3	
Total sites	222	100	49		22.0	173	78,0	40	18.0		182	81.9	
P value			0,000					0,000					
	Total			What do you know about HPV?				What do you know about HPV prevention?					
Region	*N*	%		Good knowledge		bad knowledge		Good knowledge			bad knowledge		
				*n*	%	*n*	%	*n*	%		*n*	%	
Urban	96	100		24	25,0	72	75,0	31	32,3		65	67,3	
periurban	66	100		8	12,0	58	88,0	7	10,6		59	89,4	
Rural	60	100		1	1,7	59	98,3	2	3,3		58	96.7	
Total sites	222	100		33	14.4	189	85.6	40	18.1		182	81.9	
*P* value				0,000				0,000					

P value < 0,05 = statistically significant

### Degree of knowledge about cervical cancer and HPV and their prevention

As summarized in Table 1, we observed in the survey 1 that few Bolivian women have a good knowledge about cervical cancer in the three areas (urban 35.4%, peri-urban 18.2% and rural 1.7%). Less than 32.3% of women had a good level of knowledge about cervical cancer prevention in the three regions and only 25.0, 12.0 and 1.7% of women in urban, peri-urban and rural areas, respectively, had good level of knowledge about HPV. The level of knowledge about cervical cancer, HPV infection and their prevention decreased clearly in relation to the distance from the cities (*P* value< 0,05).

### Self-sampling acceptability

The evaluation and comparison of self-sampling acceptability and confidence versus physician-sampling was carried out in one rural region (Chapare), in peripheral areas of Cochabamba city and in the central area of Cochabamba city (survey 2). Three questions were asked to evaluate the easiness, the comfort and the experienced sensation of pain using the cotton swab for the self-sampling (Tables2 and 3). More than 86.9% women in the three regions indicated that the HPV self-sampling device was easy to use (from 86.9 to 93.2%), comfortable (from 79.4 to 83.3%) and painless (64.3 to 68.9%) No significant statistical difference was found in the answers in the three areas (*P* value> 0.05). Compared to self-sampling, samples taken by physicians with vaginal speculum seemed less comfortable (from 40.5 to 71.6%), and more painful, especially in rural areas (Tables 2 and 3).

**Table 2:** Self-sampling acceptability investigated in the survey 2

Survey 2 (*N* = 221)	Was the self- sampling easy or difficult?					
Region	Total		Easy		Difficult	
	*n*	%	*n*	%	*n*	%
Urban	74	100	69	93.2	5	6.8
Periurban	63	100	56	88.9	7	11.1
Rural	84	100	73	86.9	11	13.1
Total sites	221	100	198	89.7	23	10.3
P value			0.3			
How did you feel when you took your own sample?						
	Total		Comfortable		Uncomfortable	
	*n*	%	*n*	%	*n*	%
Region						
Urban	74	100	61	82.4	13	17.6
Periurban	63	100	50	79.4	13	20.6
Rural	84	100	70	83.3	14	16.7
Total sites	221	100	181	81.7	40	18.3
P value			0.18			
How did you feel when the physician took your sample?						
	Total		Comfortable		Uncomfortable	
Region	n	%	n	%	n	%
Urban	74	100	53	71.6	21	28.4
Periurban	63	100	33	52.4	30	47.6
Rural	84	100	34	40.5	50	59.5
Total sites	221	100	120	54.3	101	45.7
P value			0.04			

*P* value < 0.05 = statistically significant

**Table 3:** Self-sampling acceptability investigated in the survey 2

Survey2 (N = 221)			Did you feel any pain when you took your own sample?					
	Total		No pain		Much pain		Litlte pain	
	*N*	%	*n*	%	*n*	%	*n*	%
Region								
Urban	74	100	51	68.9	2	2.7	21	28.4
Periurban	63	100	43	68.3	1	1.6	19	30.2
Rural	84	100	54	64.3	0	0.0	30	35.7
Total sites	221	100	148	67.2	3	1.4	70	31.4
*P* value			0.51		0.36		0.22	
Did you feel any pain when the physician took your sample?								
	Total		No pain		Much pain		Litlte pain	
	*N*	%	*n*	%	*n*	%	*n*	%
Region								
Urban	74	100	32	43.2	9	12.2	33	44.6
Periurban	63	100	22	35.0	6	9.0	35	56.0
Rural	84	100	22	26.1	7	8.3	55	65.6
Total sites	221	100	76	34.4	22	10.00	123	55.6
P value			0.26		0.72		0.02	

*P* value < 0.05 = statistically significant

### Women confidence post self-sampling procedure

In Survey 2, women were also asked for their long term screening strategy preference: physician-sampling versus self-sampling procedure (Table 4). There was no clear preference, regardless the urban, peri-urban and rural areas (*P* value> 0.05). However, in regards to the question: what test would you recommend? women from the rural areas tended to recommend the self-sampling with a significant statistical difference in the three areas (Table 4). Similarly, with regard to the question of which procedures they consider to be the best to detect cervical cancer, most women from urban area chose both procedures (51.4%), while in the peri-urban and rural area women clearly would consider samples taken by the physician the best to detect cervical cancer (63.5 and 58. 3 respectively); (*P* value < 0.05).

**Table 4:** Women’s confidence on cervical screening after self-sampling and sampling by physician (survey 2)

Survey 2 (N = 221)			Which of the two tests would you prefer to do permanently?							
	Total		Physician		Self.		Both		no answer	
Region	*N*	%	*n*	%	*n*	%	*n*	%	*n*	%
Urban	74	100	26	35.1	21	28.4	27	36.5	0.0	0.0
periurban	63	100	20	31.8	19	30.2	24	38.1	0.0	0.0
Rural	84	100	20	23.8	31	36.9	33	39.3	0.0	0.0
Total sites	221	100	66	29.9	71	32.1	84	38.0	0.0	0.0
*P* value			0.57		0.17		0.47			
Which of the two test would you recommend?										
	Total		Physician		Self.		Both		no answer	
Region	*N*	%	*n*	%	*n*	%	*n*	%	*n*	%
Urban	74	100	17	23.0	21	28.4	35	47.3	1.0	1.4
periurban	63	100	20	31.8	17	27.0	26	41.3	0.0	0.0
Rural	84	100	17	20.2	33	39.3	27	32.1	7.0	8.3
Total site	221	100	54	24.4	71	32.1	88	39.8	8.0	3.6
*P* value			0.84		0.05		0.43			
Which tests is the best to detect cervical cancer?										
	Total		Physician		Self.		Both		no answer	
Region	*N*	%	*n*	%	*n*	%	*n*	%	*n*	%
Urban	74	100	26	35.1	10	13.5	38	51.4	0.0	0.0
periurban	63	100	40	63.5	7	11.1	16	25.4	0.0	0.0
Rural	84	100	49	58.3	8	9.5	27	32.1	0.0	0.0
Total sites	84	100	115	52.0	25	11.4	81	36.6	0.0	0.0
*P* value			0.03		0.75		0.01			

Self = Self-sampling

*P* value < 0.05 = statistically significant

### Impact of HPV awareness campaign and self-sampling testing on screening coverage in the district 9 of Cochabamba

In 2015, starting in January, we promoted cervical cancer screening in District 9 health centers. Health personnel from 8 public health primary care centers in the intervention area were informed and trained about the relationship between HR-HPV and cervical cancer, prevention and molecular detection of HPV. Women attending those primary health care centers were encouraged to visit gynecologists to request the physician-collected HPV and Pap smear test. The purpose of this activity was to be able to respond to the demand for information and / or follow-up of any woman in the area requesting it.

**Fig. 1:**
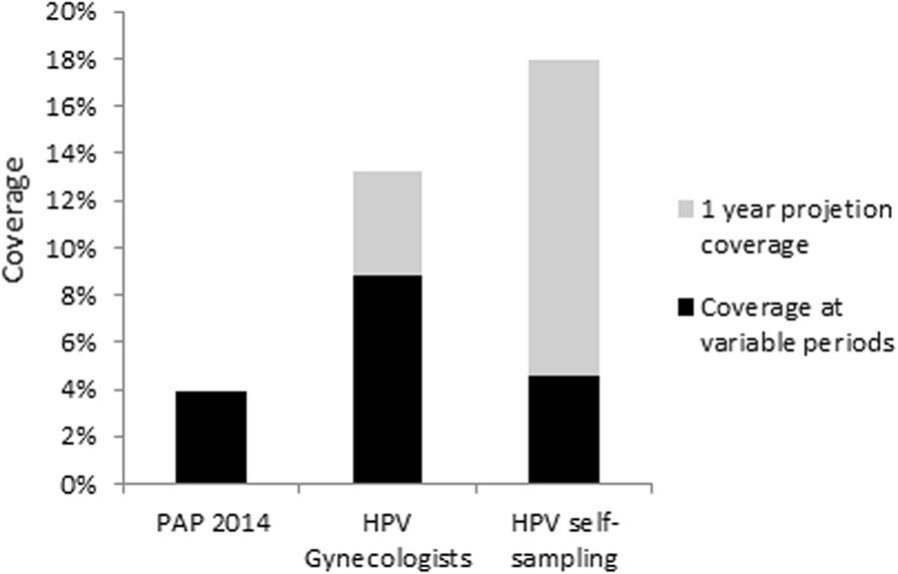
Coverage of cervical cancer screening in district 9 Cochabamba using various strategies: Papanicolaou smear during 1 year in 2014 (Pap), HPV DNA test sampled by gynecologists for 8 months in 2015, HPV DNA self-sampling for 3 months in 2015, given in black. The coverage projections for one year are given in grey

Four months later, we started campaigns (9 days distributed in a three month period), to collect self-sampling from the same population. Campaigns were performed informing and offering the self-sampling device either by visiting women from house to house or by meeting them in populated areas under a tent with isolated cubicles, providing favorable environment for self-sampling. This 9 days campaign allowed collecting 902 HPV self-sampling tests from the same population. From 902 (100%) women who underwent self-sampling, 792 (87%) samples were analyzed, 95 (12%) were HR-HPV positive, and only 13 (13.7% of women with HR-HPV) came back for follow-up Three women had a low-grade intraepithelial lesion and two had a high-grade intraepithelial lesion. The coverage calculation in District 9 was performed taking into account the population assigned to the eight health centers (19,402 women between 20 and 65 years old). The Pap smear test coverage achieved in the year 2014 was 3.9%, and coverage obtained in 2015 in three months by the HPV self-sampling test was 4.6%, making an estimated annual coverage that could achieve 18.6%, namely 4.7 fold higher with respect to the Pap coverage of the previous year (Fig. 1). The overlapping 8 month promotion of the HPV and Pap smear test in the 8 health centers of the same district, only allowed to collect approximately 849 tests, suggesting that although cervical cancer screening promotion increase screening coverage, the increase of coverage is even higher if self-sampling screening is provided. By comparison, cytology screening at District 9 health centers of Cochabamba during the 12 months of 2014 allowed to target 761 women.

It is worth noting that HR-HPV results (in all studies) were always provided 2–3 weeks after collection (by physician or self-sampling), while cytology results, when available, were mostly provided after 3 months.

## Discussion

In order to reduce the cervical cancer incidence and mortality in the department of Cochabamba in Bolivia, we aimed to develop and assess a low cost HPV self-sampling screening. We believe that this strategy could improve the poor screening programs developed in our country. Not only the HPV DNA detection rate in self-sampling has been reported to be in good agreement with physician-sampling, but also the self-sampling method seems better accepted among women [24, 25]. It could therefore enhance cervical cancer screening program coverage, a primary prevention strategy in countries with low resources [26].

In our study, in the Survey 1, we confirmed that the level of knowledge about cervical cancer and HPV, in the three geographical areas, was low, diminishing in function of the distance between the area of residence and the capital city (urban area), and of the education level (the null hypothesis is rejected). A study in Cameroon mentioned that education about HPV and cervical cancer is associated to a greater acceptability towards the HPV self-sampling test. The provided information had a positive impact on the patient’s concerns, about her ability to perform the test [27]. Therefore, not only reduced access but also lack of knowledge about these issues could potentially participate to the low cervical cancer screening coverage observed in the Bolivian remote areas. This concern should therefore always be addressed in future screening campaign in Bolivia.

According to the results obtained in the survey 2, most of the women found, in the three geographic areas, self-sampling easy, comfortable and without pain. The level of the self-sampling acceptance was thus good, allowing to consider it as a promising screening tool to break sociocultural barriers. A similar result was observed in a study performed on three different populations (Uganda, Nicaragua and India) as 75% women found that self-sampling was easy and 90% found it very comfortable [28]. However, we observed in survey 2 that there were no clear preference to use it as a form of cervical sampling for the detection of cervical cancer in the three areas, despite a tendency in the rural area to recommend it. This result denoted a distrust in the self-collected vaginal sample. Other studies concluded similarly and found that, despite great acceptability for self-sampling, many women were concerned about test accuracy to detect the lesions and even more concerned by the risk of not having taken correctly the sample [28, 29]. This result was unexpected as in our previous study in Bolivia, in a population of women having previous experience with Pap test at 76%, self-sampling was clearly preferred. This striking difference between this previous study and the present study might be due to the fact that in the previous study women were not attending health center and could have therefore more hesitation to visit a gynecologist [22], while in our study (Phase 2) women were already attending health center. The context and the population in which both studies were performed were thus clearly different and it could have had some influence on the collected responses.

A meta-analysis in which six studies were reviewed about self-sampling acceptability revealed that the majority of women interviewed preferred the self-sampling test which did not require a gynecological examination [25]. The vaginal self-sampling could therefore potentially increase cervical cancer screening coverage [29].

In our third study, assessing self-sampling on cervical cancer screening coverage in a peri-urban area (9th District), we observed up to a 4.7 fold higher screening coverage compared to the previous year Pap coverage. Similarly, another study in South America mentioned a 4 fold increase in screening coverage using self-sampling [30] and similar results were recently obtained in other countries of Latino America [31, 32]. Although, in our third phase study, we did not register the number of women rejecting self-sampling, we observed during the campaigns that most women were very likely to use it after a detailed explanation about the HPV infection and its relationship with cervical cancer. In agreement with our results obtained in the Survey 1 and 2, we believe that intervention with this strategy (primary information and awareness transfer before offering self-sampling) will provide more all women confidence to perform self-sampling, a strategy that could counteract their lack of self-assurance and even their lack of knowledge. Many women, not screened in one of the 8 health centers assigned to the 9th district, were screened by the vaginal self-sampling test. Although awareness campaigns were associated to the self-sampling offer and present in the health centers, these campaigns were not similar. It is therefore possible that this co-intervention was necessary to achieve a successful coverage. Campaigns performed through visits at home or in populated areas to offer only self-sampling were transitorily done by unsubsidized volunteers, while campaigns for Pap and HPV detection performed in health centers were permanently present under the form of posters and by health personnel sensitization. It is therefore clear, that further studies will be necessary to aim at maintaining similar good results with same limited resources. The follow-up of the HR-HPV positive patients was however low and unsatisfactory. This low level of follow-up was not due to a lack of health personnel, but mainly due to a lack of women concern (as the number of women going to health centers for follow-up was very low). Further investment in women encouragement should be provided in screening campaigns to improve the follow-up rate and keep the benefit of a successful coverage rate. It will be interesting to evaluate which barriers and factors are associated to the low observed follow-up rate. Although self-sampling screening could be beneficial to reach a higher screening coverture rate, the follow-up strategy should take into account the difficulty to motivate women to visit a health center. A screen-and-treat strategy (a screening based on HPV detection or VIA, followed by cryotherapy or LEEP, if necessary) could therefore be even more beneficial for this population. However, the cost, sensitivity and specificity of the strategy should be carefully evaluated.

If self-sampling would be routinely used in Bolivia, additional infrastructures and guidelines will be needed, especially to provide clear information, among others to prevent insecurity feelings during samplings, to increase appropriated patient engagement, to improve the follow-up rate, but also to reduce the risk of patient anxiety after a positive result diagnosis transfer [33].

## Conclusions

In conclusion, the self-sampling is well accepted by the Bolivian surveyed women, regardless of demographic differences. This suggested that in Bolivia also, cervical cancer detection coverage can be expanded using this form of cervical sampling (self-sampling). Knowledge about cervical cancer and HPV should be optimized to improve cervical cancer prevention programs. This aspect is fundamental to improve confidence and motivation of women performing self-sampling Studies to assess follow-up efficacy in regards to screening strategy should be further performed.

## Additional Files

Additional file 1: Survey 1: Level of knowledge about HPV and cervical cancer. Multiple choice questionnaire in the three geographical areas of study to determine the degree of knowledge about cervical cancer and HPV. (PDF 414 kb)

Additional file 2: HPV self-sampling kit. Self-sampling kit consisting of a cotton swab and a glass slide for cervical / vaginal sampling (TIF 222 kb)

## Abbreviations

DNA: Deoxyribonucleotide acid
HPV: Human papillomavirus
HR-HPV: High-risk human papillomavirus
Pap: Papanicolaou
PCR: Polymerase chain reaction
VIA: Visual Inspection with Acetic Acid

## Footnotes

1 Cervical cancer incidence and mortality rates in Bolivia were roughly estimated from regional data.
