# Supplementary material for: Evaluation of the self-sampling for cervical cancer screening in Bolivia
Source: BMC Public Health. 2019 Jan 17;19:80. doi: 10.1186/s12889-019-6401-5 (PMC6337790; doi:10.1186/s12889-019-6401-5)
Supplement: Supplementary file 1 — Survey 1: Level of knowledge about HPV and cervical cancer. Multiple choice questionnaire in the three geographical areas of study to determine the degree of knowledge about cervical cancer and HPV. (PDF 414 kb) [file 12889_2019_6401_MOESM1_ESM.pdf]

KNOWLEDGE QUESTIONS

1. –How old are you?

a. - 20 to 29

b. - 30 to 39

c. - 40 to 49

d. - 50 to 59

e. - More than 59

2. - What level of education did you reach?

a. - Primary or lower

b. - High school

c. - University

d. - Other, please specify...

e. - No response

3. - What do you know about cervical cancer? (Check all that apply)

a. - It is a cancer that most often affects middle-aged or older women

b. - It is a cancer that mostly affects younger women

c. - It is a cancer that affects the lower genital tract of women

d. - Persistent infection with some types of Human Papilloma Virus (HPV) causes cervical cancer

e. - Cervical cancer is the second most common cancer among women worldwide

f. - Only sexually active women can develop cervical cancer

g. - Do not know

4. - What do you know about cervical cancer prevention? (Check all that apply)

a. - Regular search or screening can prevent cancer almost entirely

b. - Doing my Pap test

c. - Doing my HPV test

d. - Receiving vaccine against HPV

e. - The danger of getting cervical cancer decreases if my partner uses condoms during sexual intercourse.

f. - Do not know

g. - No response

5. - What do you know about the Human Papilloma Virus (HPV)? (Check all that apply)

a- - HPV is a sexually transmitted virus

b. - Not all HPV infections can lead to cancer

c. - Both, men and women can contract HPV sexually

d. - A positive HPV test is usually not followed by symptoms

e. - Others, please specify

f. - Do not know

g.- No response

6.-What do you know about Human Papilloma Virus (HPV) prevention? (Check all that apply)

a. - Condom use when having sexual intercourse can decrease the probability of acquiring HPV

b. - The use of condoms helps avoid sexually transmitted diseases such as HIV, chlamydia, fungal vaginal infection, etc.

c. - Having only one or a few partners can reduce the probability of getting infected by HPV

d. - Others, please specify...

e. - Do not know
