# Supplementary figures and images for: Evaluation of the self-sampling for cervical cancer screening in Bolivia
Source: BMC Public Health. 2019 Jan 17;19:80. doi: 10.1186/s12889-019-6401-5 (PMC6337790; doi:10.1186/s12889-019-6401-5)

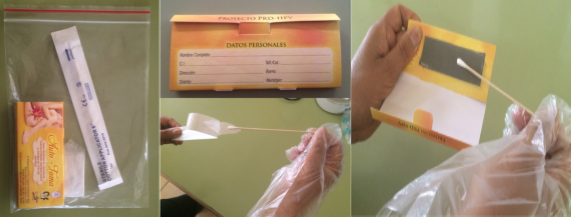

Supplement: Supplementary file 2 — HPV self-sampling kit. Self-sampling kit consisting of a cotton swab and a glass slide for cervical / vaginal sampling (TIF 222 kb) [file 12889_2019_6401_MOESM2_ESM.tif]
